# Peroxisome Proliferator-Activated Receptor-*α* Activation Decreases Mean Arterial Pressure, Plasma Interleukin-6, and COX-2 While Increasing Renal CYP4A Expression in an Acute Model of DOCA-Salt Hypertension

**DOI:** 10.1155/2011/502631

**Published:** 2011-12-07

**Authors:** Dexter L. Lee, Justin L. Wilson, Rong Duan, Tamaro Hudson, Ahmed El-Marakby

**Affiliations:** ^1^Department of Physiology and Biophysics, College of Medicine, Howard University, 520 W Street, NW, Washington, DC 20059, USA; ^2^Department of Medicine, Cancer Center, Howard University, Washington, DC 20059, USA; ^3^Department of Oral Biology and Department of Pharmacology, Georgia Health Sciences University, Augusta, GA 30912, USA

## Abstract

Peroxisome proliferator-activated receptor-alpha (PPAR-**α**) activation by fenofibrate reduces blood pressure and sodium retention during DOCA-salt hypertension. PPAR-**α** activation reduces the expression of inflammatory cytokines, such as interleukin-6 (IL-6). Fenofibrate also induces cytochrome P450 4A (CYP4A) and increases 20-hydroxyeicosatetraenoic acid (20-HETE) production. This study tested whether the administration of fenofibrate would reduce blood pressure by attenuating plasma IL-6 and renal expression of cyclooxygenase-2 (COX-2), while increasing expression of renal CYP4A during 7 days of DOCA-salt hypertension. We performed uni-nephrectomy on 12–14 week old male Swiss Webster mice and implanted biotelemetry devices in control, DOCA-salt (1.5 mg/g) treated mice with or without fenofibrate (500 mg/kg/day in corn oil, intragastrically). Fenofibrate significantly decreased mean arterial pressure and plasma IL-6. In kidney homogenates, fenofibrate increased CYP4A and decreased COX-2 expression. There were no differences in renal cytochrome P450, family 2, subfamily c, polypeptide 23 (CYP2C23) and soluble expoxide hydrolase (sEH) expression between the groups. Our results suggest that the blood pressure lowering effect of PPAR-**α** activation by fenofibrate involves the reduction of plasma IL-6 and COX-2, while increasing CYP4A expression during DOCA-salt hypertension. Our results may also suggest that PPAR-**α** activation protects the kidney against renal injury via decreased COX-2 expression.

## 1. Introduction

Peroxisome proliferator-activated receptor-alpha (PPAR-*α*) activation has been implicated in blood pressure regulation and different models of hypertension [1–6]. During DOCA-salt-induced hypertension, activation of PPAR-*α* increases renal tubular cytochrome P450 4A (CYP4A) expression, thereby increasing 20-hydroxyeicosatetraenoic acid (20-HETE) production which affects sodium retention and reduces blood pressure. PPAR-*α* deficiency blocks these effects, suggesting the dependency on PPAR-*α* [6]. PPAR-*α* has been found to colocalize with arachidonate CYP450 4A enzymes in the renal proximal tubule and P450 4A monooxygenases are known PPAR-*α* target genes [7]. A previous report also demonstrates PPAR-*α* activation exerts protective actions during DOCA-salt-induced hypertension via mechanism that involves nitric oxide production and/or inhibition of NAD(P)H oxidase activity and reduced generation of reactive oxygen species (ROS) [8]. PPAR-*α* activation has also been shown to increase nitric oxide generation and promote renal excretion of Na^+^ through reduced Na^+^-K^+^ ATPase in the proximal tubule [9]. Therefore, PPAR-*α* plays an important role of regulating blood pressure during hypertension.

Peroxisome proliferator-activated receptor-alpha (PPAR-*α*) activation plays a major role in the regulation of vascular inflammation [10, 11]. Proinflammatory cytokines have been implicated in the pathogenesis of hypertension [12–14]. PPAR-*α* deficiency has resulted in elevated expression of nuclear factor- *κ*B (NF-*κ*B), an important pro-inflammatory transcription factor [15]. Fenofibrate, a PPAR-*α* agonist, has been shown to decrease monocyte release of numerous cytokines, including interleukin-6 (IL-6), monocyte chemoattractant protein-1 (MCP-1), lymphocyte release of interleukin-2, interferon-*γ*, and tumor necrosis factor-*α* (TNF-*α*), which was accompanied by a decrease in plasma C-reactive protein levels [16]. IL-6 has been shown to play a major role in the hypertension of human and animal models [12, 17, 18]. DOCA-salt hypertension causes an increase in renal IL-6, TNF-*α*, MCP-1, endothelin-1, and reactive oxygen species [19]. Previous results also demonstrate that five weeks of administering fenofibrate significantly attenuated IL-6, cyclooxygenase-2 (COX-2), vascular cell adhesion molecule-1, and MCP-1, reduced myocardial fibrosis, and prevented the development of diastolic function during DOCA-salt treatment. It was concluded that the five-week treatment with fenofibrate was mediated partly by prevention of inflammatory mediators through the NF-kappa-B pathway [20]. Recombinase-activating gene (RAG-1) knockout mice, which lack both T and B lymphocytes, show a blunted hypertensive response during DOCA-salt hypertension [21]. A previous study also demonstrates plasma levels of IL-6 are elevated on day 7 of DOCA-salt-induced hypertension [22]. Therefore, the goal of this study was to determine if PPAR-*α* activation during 7-day treatment of DOCA-salt lowered blood pressure through anti-inflammatory mechanisms by reducing COX-2 expression in the kidney and lowering plasma IL-6, as well as increasing renal CYP4A expression.

## 2. Methods

### 2.1. Telemetry

Procedures involving animals were approved by the Animal Care and Use Committee of Howard University. The experiments were conducted on 12-to 14-week-old Swiss Webster (28–32 g) male mice that were divided into three groups (control, DOCA-salt, and DOCA-salt + fenofibrate, *n* = 7–8 per group). The animals were anesthetized using isoflurane, and biotelemetry transmitter devices (PA-C10, Data Sciences, St. Paul, MN) were implanted using aseptic technique. The catheter was implanted in the left carotid artery through an incision in the vessel wall made with a custom-shaped 27.5-gauge needle. The body of the transmitter was tunneled subcutaneously above the right shoulder and secured above the scapula. The left kidney was removed from all mice through a flank incision during the transmitter surgery. All incisions were infiltrated with 1% lidocaine and mice were placed in warm cages to recover following surgery. The mice were transferred to a light- and temperature-controlled room in the animal facilities and were housed individually in standard mouse cages with tap water and standard rodent chow available *ad libitum*. The mice were given 7-days to recover from surgery before control measurements were made. Baseline mean arterial pressure and locomotor activity were collected for 3 days. After the collection of baseline data, a DOCA pellet (1.5 mg/g, Innovative Research of America, Sarasota, FL) was implanted subcutaneously for seven-days. The dose of DOCA treatment was similar to the dosage used in previous studies [6, 22]. All mice were given a drinking solution of 1% NaCl and 0.1% KCl. Mice treated with fenofibrate were given a dose of 500 mg/kg/day in corn oil, intragastrically (ig). A previous report used the same dose for a different PPAR-*α* agonist (clofibrate) during DOCA-salt hypertension [6]. Mean arterial pressure and locomotor activity were recorded 19-hours/day for a total of 10 days. On day 7 of DOCA-salt, mice were anesthetized with isoflurane, and blood, heart, and kidney were harvested.

### 2.2. Plasma IL-6 Concentration

Plasma IL-6 concentrations were measured by enzyme immunoassay (R&D Systems, Minneapolis, MN) from terminal femoral artery blood samples obtained on day 7 of DOCA treatment. Animals from all groups were included on the same assay plate to control for interassay variability.

### 2.3. Urinary Albumin Concentration

Albumin concentration was measured from a one day timed urine collection on control day 7, DOCA and DOCA + fenofibrate day 7, using an Albuwell murine microalbuminuria ELISA (Exocell).

### 2.4. Mouse Cytokine Array

A mouse cytokine array (R&D Systems, Minneapolis, MN) was used to determine protein expression of the inflammatory cytokines. Plasma samples from the control, DOCA-salt, and DOCA + fenofibrate were collected using heparin as an anticoagulant. Plasma samples were centrifuged for 20 minutes at approximately 2000 × g within 30 minutes of collection. Plasma samples were combined with array buffers, aspirated over the well multidishes and incubated overnight. After multiple washes, well multi-dishes were incubated with Streptavidin-HRP. Images were analyzed by using the NIH imaging software, Image J. Pixel density for each inflammatory cytokine was normalized against the respective positive control on each well multidish. Data are expressed as a percent change.

### 2.5. Western Blot

Whole kidney homogenized protein samples (50 *μ*g) were separated by sodium dodecyl sulfate-polyacrylamide gel electrophoresis on a 10% Tris-glycine gel, and proteins were transferred electrophoretically to a PVDF membrane. Non-specific binding sites were blocked by incubating the blots overnight at 4°C in a Tris-NaCl buffer (TBS) containing 5% nonfat dry milk and 0.1% Tween 20. The primary antibodies used are rabbit CYP2C23, CYP4A, sEH, (1 : 1000; Santa Cruz, CA) and COX-2 (1 : 1000, Cayman Chemical). The blots were then washed in a TBS-0.1% Tween and incubated with the secondary antibody goat anti-rabbit 1 : 5000 and goat anti-mouse 1 : 10000 for *β*-actin) conjugated to horseradish peroxidase for 1 hour and washed. Detection was accomplished using enhanced chemiluminescence western blotting, and band intensity was measured densitometrically and the values were normalized to *β*-actin.

### 2.6. Statistical Analysis

Mean arterial pressure data were analyzed with a repeated measure ANOVA. Significant *F*-test from the ANOVA at *P* < 0.05 was followed by post hoc comparisons using the Newman-Keuls multiple range test. A one-way ANOVA was used to analyze additional parameters with the Krusal-Wallis post hoc test. Significance was considered at *P* < 0.05.

## 3. Results

**Figure 1:**
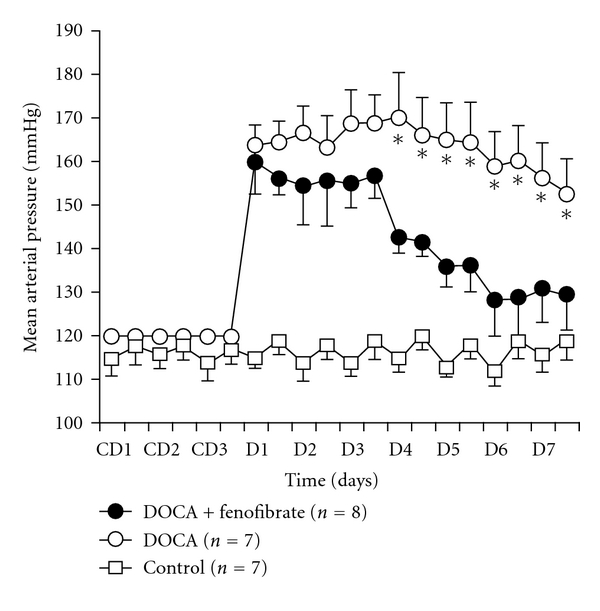
Mean arterial pressure during a seven-day treatment period in control mice, DOCA-salt-treated (1.5 mg/g) group and a DOCA + fenofibrate-treated (500 mg/kg/day) group. C: control days, D: DOCA salt days (*P < 0.05).

**Figure 2:**
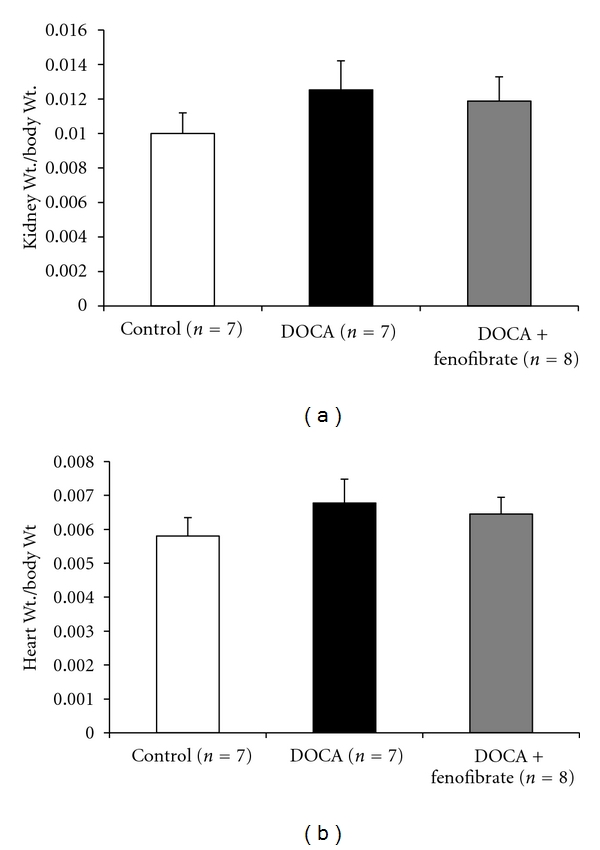
Kidney- and heart-weight-to-body-weight ratios in control, DOCA-salt-treated, and DOCA + fenofibrate-treated mice.

Figure 1 shows mean arterial pressure of control, DOCA-salt and DOCA-salt + fenofibrate treated mice. Baseline mean arterial pressure was not different between the groups. DOCA-salt treatment had a significant hypertensive effect when compared to control mice. The initial increase in mean arterial pressure was also similar in the DOCA and DOCA-salt + fenofibrate groups. The administration of fenofibrate resulted in a significant decrease in mean arterial pressure on days 4–7 of DOCA-salt treatment. The mean arterial pressure in the DOCA-salt + fenofibrate treated group returned to near baseline levels by day 7 of DOCA-salt treatment. DOCA-salt + fenofibrate and control mice MAP were not different on days 6-7.  Figure 2 shows the kidney and heart weight to body weight ratios in control, DOCA-salt treated and DOCA-salt + fenofibrate treated mice. Although DOCA-salt slightly increased and DOCA-salt + fenofibrate slightly decreased the kidney and heart weights to body weight ratios, there were no significant differences between the kidney and heart weight to body weight ratios of the control, DOCA-salt and DOCA-salt + fenofibrate treated groups. There were also no significant differences in locomotor activity of the control, DOCA-salt and DOCA-salt + fenofibrate treated groups (data not shown). 

**Figure 3:**
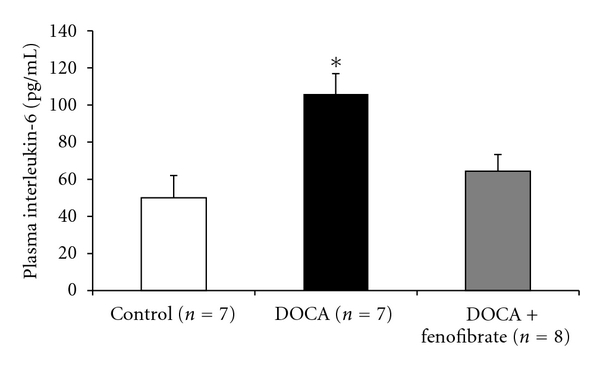
Plasma interleukin-6 on day seven of DOCA-salt hypertension in control, DOCA and DOCA + fenofibrate-treated mice (*indicates DOCA significantly increased plasma IL-6 when compared to control and DOCA + fenofibrate; *P* < 0.05).

**Figure 4:**
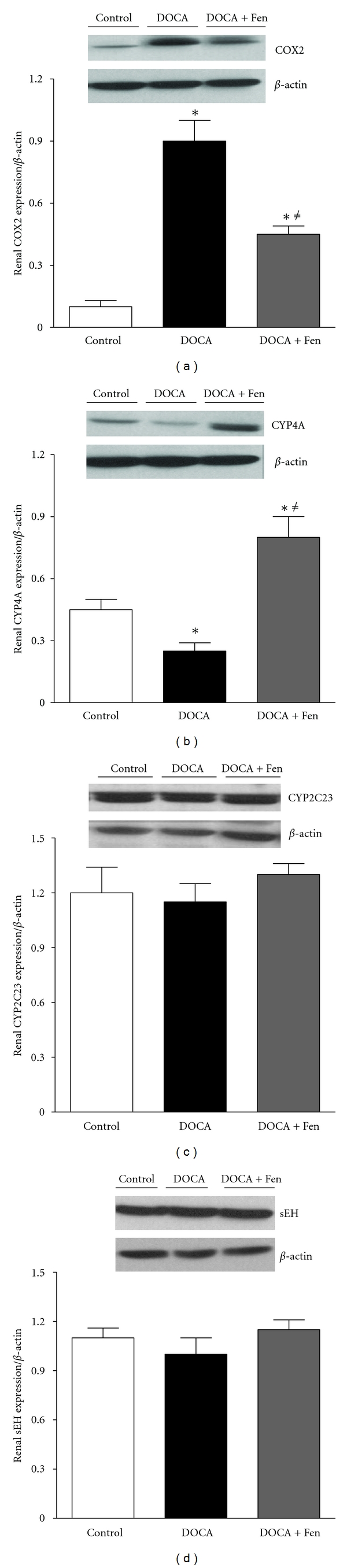
Western blot expression and analysis of (a) COX-2, (b) CYP4A, (c) CYP2C23, and (d) sEH in whole-kidney homogenates taken from control, DOCA, and DOCA + fenofibrate mice (*indicates DOCA + fenofibrate significantly reduced COX-2 expression when compared to DOCA alone. ^*≠*^indicates DOCA + fenofibrate COX-2 expression was significantly higher than the control group. *indicates DOCA and DOCA + fenofibrate have significantly different renal CYP4A expression when compared to control. ^*≠*^indicates DOCA + fenofibrate renal expression of CYP4A is significantly increased when compared to DOCA (*P* < 0.050)).

Our initial investigation of plasma cytokines during DOCA-salt treatment involved the utilization of a mouse cytokine array to determine changes in plasma samples during the seven-day period of DOCA-salt hypertension. The preliminary results from the mouse cytokine array revealed a 30 ± 7% increase in plasma expression of IL-6 in DOCA-salt treated mice when compared to the DOCA-salt + fenofibrate group. Plasma expression of IL-6 in control and DOCA-salt + fenofibrate treated groups were similar (data not shown). We did not see significant differences in MCP-1 or TNF-*α* plasma expression between the DOCA-salt and DOCA-salt + fenofibrate groups. Therefore, we later performed an IL-6 enzyme immunoassay to determine the concentration of IL-6 in the plasma of all groups. In Figure 4, our plasma IL-6 results on day 7 of DOCA-salt hypertension illustrate that fenofibrate caused a significant reduction in plasma IL-6, when compared to DOCA-salt alone. Plasma IL-6 levels were not different between the control and DOCA-salt + fenofibrate treated groups (Figure 3).

Whole kidney homogenates were used to determine COX-2, CYP4A, soluble expoxide hydrolase and CYP2C23 expression during DOCA-salt treatment. Fenofibrate treatment during DOCA-salt hypertension significantly reduced COX-2 renal expression when compared to DOCA-salt alone. However, fenofibrate did not return COX-2 renal expression back to control levels. Fenofibrate treatment also significantly increased CYP4A when compared to DOCA-salt and control groups. No differences were observed in the renal expression of sEH and CYP2C23 between the groups (Figure 4). 

**Figure 5:**
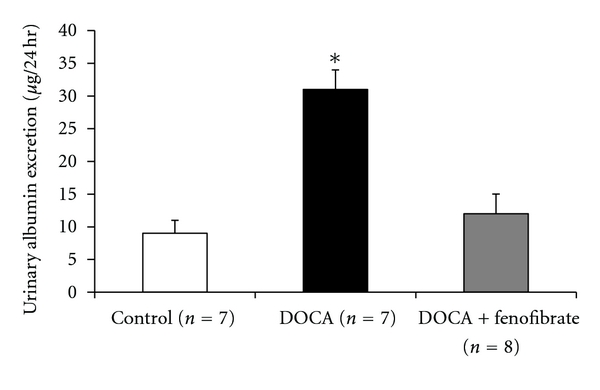
Urinary albumin excretion on day seven in control, DOCA, and DOCA + fenofibrate-treated mice. *indicates DOCA albumin excretion is significantly different from control and DOCA + fenofibrate-treated mice.

Renal injury was assessed on day 7 of DOCA-salt hypertension by measuring 24-hour albumin excretion. DOCA-salt hypertension significantly increased microalbuminuria on day 7 and fenofibrate significantly reduced albumin excretion. There was no significant difference of albumin excretion between control and DOCA + fenofibrate-treated mice (Figure 5).

## 4. Discussion

The results from this study demonstrate that the PPAR-*α* agonist fenofibrate causes a significant reduction in blood pressure during a 7-day model of DOCA-salt hypertension by significantly reducing plasma IL-6 and increasing CYP4A expression in the kidney. We further show that fenofibrate also caused a significant reduction in renal COX-2 expression. Our results also demonstrate that in our 7-day model of DOCA-salt hypertension, fenofibrate did not cause a significant reduction in heart- and kidney-weight-to-body-weight ratios. Therefore, on the basis of these and previous findings, we hypothesize that the activation of PPAR-*α* with the agonist fenofibrate, during a 7-day model of DOCA-salt hypertension causes a reduction in blood pressure via an anti-inflammatory and direct renal mechanisms. Previous results demonstrate that bezafibrate [23] and clofibrate [6], inducers of CYP4A and 20-HETE have been shown to decrease blood pressure in DOCA-salt hypertensive mice. In addition, the COX-2 inhibitor, SC58236, reduced renal inflammation and injury of diabetic DOCA-salt hypertensive rats independent of blood pressure lowering effects [24]. In the current study, we extend the blood pressure lowering observations of the inducers of CYP4A and 20-HETE, to demonstrate that fenofibrate may also reduce blood pressure through IL-6-dependent mechanism.

Previous results demonstrate that plasma IL-6 is significantly elevated on day 7 of DOCA-salt hypertension and decreases to baseline levels on day 14 with no significant reduction in blood pressure. In addition, DOCA-salt treatment in IL-6 KO mice demonstrated that mineralocorticoid hypertension does not require IL-6 [22]. Therefore, it is hypothesized that increases in plasma IL-6 are not needed for blood pressure elevation after day 7 of DOCA-salt hypertension. The present study goal was to determine if the increase in plasma IL-6 on day seven of DOCA-salt hypertension plays a major role in the blood pressure elevation. Our goal was to also determine if PPAR-*α* activation would decrease inflammatory markers and promote renal regulation of blood pressure through a CYP4A and 20-HETE pathway. Our results suggest that during a 7-day model of DOCA-salt hypertension, IL-6 plays a major role in blood pressure regulation. Additional studies are needed to determine which inflammatory markers are elevated during a 7-day model of DOCA-salt hypertension in the absence of plasma IL-6. Results from our cytokine array suggest that plasma levels pro-inflammatory cytokines TNF-*α* and MCP-1 were not significantly different between DOCA-salt and DOCA-salt + fenofibrate groups and may not contribute to blood pressure differences in our acute model of DOCA-salt hypertension. In addition, future studies are needed to determine if the role of IL-6 on blood pressure during DOCA-salt hypertension is different in Swiss Webster mice used in this study and C576BL6 used in previous studies [19, 22].

A previous study demonstrates DOCA-salt hypertension causes an increase in inflammatory markers, including renal TNF-*α*, MCP-1, and IL-6 [19]. Previous results also demonstrate that COX-2 expression is increased in a model of DOCA-salt hypertension [25, 26]. In an experimental model of diabetes and DOCA-salt hypertension, inhibition of COX-2 expression decreases potential mediators of glomerular and tubulointerstitial injury and also decreases biochemical, functional, and structural markers of renal injury [24]. PPAR-*α* activation has also been shown to blunt renal damage that is characteristic of DOCA-salt hypertension [8]. Previous results also show that COX-2 expression increases during DOCA-salt hypertension and mediates production of factors that enhances vasoconstriction [25]. Our results demonstrate that PPAR-*α* activation with fenofibrate caused a significant decrease in renal COX-2 expression. Our results may also suggest that PPAR-*α* activation during a seven-day model of DOCA-salt hypertension decrease markers of renal injury.

Cytochrome P450 (CYP) 4A gene catalyzes the synthesis of 20-HETE. In renal tubular segments, 20-HETE is an important mediator in the regulation of sodium transport. Previous results demonstrate that increased 20-HETE production by PPAR-*α* activation is also involved in the regulation of blood pressure and sodium retention in a DOCA-salt model. More specifically, sodium retention was associated with reduced renal 20-HETE production [6]. The previous report also demonstrates that treating DOCA-salt mice with clofibrate, a PPAR-*α* agonist, attenuated the increase in mean arterial pressure and cumulative sodium balance while increasing 20-HETE production and renal CYP4A expression [6]. Previous reports also demonstrate that PPAR-*α* agonists benzofibrate [27] and fenofibrate [3] increases renal CYP4A expression in mice and cause a significant reduction in blood pressure during DOCA-salt and angiotensin II hypertension, respectively. In the present study, our results also suggest that a seven-day treatment of DOCA-salt increases renal CYP4A expression during fenofibrate treatment and cause, antihypertensive mechanisms that may involve an increase in 20-HETE production.

 Soluble epoxide hydrolase (sEH) contributes to hypertension [28], inflammation, and renal injury [29]. Reduction in renal inflammation and injury was observed in a 21-day model of DOCA-salt hypertension with the use of a sEH inhibitor, demonstrating that the C-terminal hydrolase domain of the sEH enzyme is responsible for renal protection with DOCA-salt hypertension [29]. Our results demonstrate that treatment with the PPAR-*α* agonist, fenofibrate, did not reduce sEH during a seven-day model of DOCA-salt hypertension. Although CYP2C23 is the main epoxygenase that drive epoxyeicosatrienoic acids production to produce vasodilatory metabolites that are antihypertensive and anti-inflammatory effects [30], we did not observe a significant effect of fenofibrate on renal CYP2C23 expression in our seven-day model of DOCA salt hypertension. Our results and previous studies suggest sEH and CYP2C23 contribute to the hypertension after 7-days of DOCA-salt treatment.

In summary, the current study demonstrates that PPAR-*α* agonist fenofibrate causes a reduction in blood pressure during a 7-day model of DOCA-salt hypertension by reducing renal COX-2 and increasing CYP4A expression while decreasing plasma levels of IL-6. The ability of fibrates to induce CYP4A and increased 20-HETE production could be a potential treatment for salt-sensitive hypertension. In addition, PPAR-*α* modulates the renin-angiotensin-aldosterone system, a major regulator of systemic blood pressure and interstitial fluid volume by transcriptional control of renin, angiotensin, angiotensin converting enzyme, and angiotensin II receptor [31]. Therefore, PPAR-*α* activation can cause a significant reduction in blood pressure through direct renal and anti-inflammatory mechanisms during a short-term model of DOCA-salt hypertension.
